# Risk assessment in COVID‐19: Prognostic importance of cardiovascular parameters

**DOI:** 10.1002/clc.23883

**Published:** 2022-07-05

**Authors:** Monika Zdanyte, Peter Martus, Jeremy Nestele, Alexander Bild, Lars Mizera, Andreas Glatthaar, Álvaro Petersen Uribe, Frederic Emschermann, Jessica‐Kristin Henes, Tobias Geisler, Karin Müller, Meinrad Gawaz, Dominik Rath

**Affiliations:** ^1^ Department of Cardiology and Angiology University Hospital Tübingen Tübingen Germany; ^2^ Institute for Clinical Epidemiology and Applied Biostatistics University Hospital Tübingen Tübingen Germany; ^3^ Eberhard‐Karls‐Universität Tübingen, Geschwister‐Scholl‐Platz Tübingen Germany

**Keywords:** cardiovascular disease, COVID‐19, risk assessment

## Abstract

**Background:**

Cardiovascular risk factors and comorbidities are highly prevalent among COVID‐19 patients and are associated with worse outcomes.

**Hypothesis:**

We therefore investigated if established cardiovascular risk assessment models could efficiently predict adverse outcomes in COVID‐19. Furthermore, we aimed to generate novel risk scores including various cardiovascular parameters for prediction of short‐ and midterm outcomes in COVID‐19.

**Methods:**

We included 441 consecutive patients diagnosed with SARS‐CoV‐2 infection. Patients were followed‐up for 30 days after the hospital admission for all‐cause mortality (ACM), venous/arterial thromboembolism, and mechanical ventilation. We further followed up the patients for post‐COVID‐19 syndrome for 6 months and occurrence of myocarditis, heart failure, acute coronary syndrome (ACS), and rhythm events in a 12‐month follow‐up. Discrimination performance of DAPT, GRACE 2.0, PARIS‐CTE, PREDICT‐STABLE, CHA2‐DS2‐VASc, HAS‐BLED, PARIS‐MB, PRECISE‐DAPT scores for selected endpoints was evaluated by ROC‐analysis.

**Results:**

Out of established risk assessment models, GRACE 2.0 score performed best in predicting combined endpoint and ACM. Risk assessment models including age, cardiovascular risk factors, echocardiographic parameters, and biomarkers, were generated and could successfully predict the combined endpoint, ACM, venous/arterial thromboembolism, need for mechanical ventilation, myocarditis, ACS, heart failure, and rhythm events. Prediction of post‐COVID‐19 syndrome was poor.

**Conclusion:**

Risk assessment models including age, laboratory parameters, cardiovascular risk factors, and echocardiographic parameters showed good discrimination performance for adverse short‐ and midterm outcomes in COVID‐19 and outweighed discrimination performance of established cardiovascular risk assessment models.

## 1 INTRODUCTION

Since the beginning of the pandemic, evidence has been mounting that SARS‐CoV‐2 infection is frequently accompanied by cardiovascular complications. Arterial hypertension, hyperlipidemia, diabetes, history of or current smoking, and history of coronary artery disease (CAD) are the comorbidities most often identified in COVID‐19 patients.^1^ Pre‐existing cardiovascular disease (CVD) is associated with disease severity and higher all‐cause mortality (ACM) in COVID‐19 patients.^2^, ^3^ Reported cardiovascular complications associated with COVID‐19 include myocardial injury, defined by elevated troponin I (TnI), myocarditis, acute myocardial infarction (AMI), heart failure and arrhythmias.^4^

Due to enormous pro‐thrombotic and fibrinolytic imbalance in COVID‐19, a significantly higher prevalence of thromboembolic complications compared to other critical illnesses is reported.^5^ Pulmonary embolism (PE) and deep vein thrombosis (DVT) are the most common COVID‐19‐associated vascular complications.^6^, ^7^ According to previous studies, PE occurs more frequently than DVT.^6^, ^7^ Therefore, PE is suggested to develop rather from local immunothrombotic processes in pulmonary vasculature (microangiopathy vs. macroangiopathy) than from embolic complications due to DVT.^8^

Arterial thrombotic events seem to be less common than venous thromboembolism (VTE). According to Klok et al.,^6^ arterial thrombotic events occurred in 3.7% of critically ill COVID‐19 patients. Bilaloglu et al.^9^ reported an 8.9% prevalence of AMI in 3334 intensive care unit (ICU) and non‐ICU patients.

A variety of risk assessment models for prediction of mortality and/or ICU treatment among COVID‐19 patients exist, for example, the Quick COVID‐19 Severity Index (qCSI), COVID‐GRAM, 4C Mortality Score.^10^, ^11^, ^12^ A score by Galloway et al. and The Veterans Health Administration COVID‐19 (VACO) Index are among the few ones which include cardiovascular risk factors (CVRFs), for example, arterial hypertension and diabetes mellitus.^13^, ^14^ Risk assessment models considering cardiac biomarkers or echocardiographic parameters are still rare. We recently showed that a multivariable model including N‐terminal prohormone of brain natriuretic peptide (NT pro‐BNP), TnI, and d‐dimer showed good discrimination performance for mechanical ventilation and ACM in a 30‐day follow‐up period. However, echocardiographic parameters failed to discriminate between favorable and adverse outcomes in COVID‐19 patients.^15^

Previous data strongly suggest an association between cardiovascular burden and increased mortality in COVID‐19. Hence, we sought to examine whether existing risk assessment models, originally established to predict the risk of thromboembolic and bleeding complications in CVD patients, may help to identify COVID‐19 patients at high risk for an unfavorable course of disease. We evaluated GRACE 2.0 score which was developed on the basis of a global registry including 102.341 acute coronary syndrome (ACS) patients and aims to predict the risk of ACM up to 3 years after an ACS as well as the risk of myocardial infarction (MI) after 1 year.^16^, ^17^ We also included the well‐known CHA_2_DS_2_‐VASc score assessing the risk of stroke and thromboembolism in patients with atrial fibrillation (AF).^18^ Several scores dealing specifically with CAD patients were also evaluated, for example, DAPT, PREDICT‐STABLE, and PARIS‐CTE which help decide on the duration of dual antiplatelet therapy (DAPT) following a percutaneous coronary intervention (PCI) aiming to lower the occurrence of thromboischemic events without increasing the bleeding risk.^19^, ^20^, ^21^ The latter can be assessed by PARIS‐MB and PRECISE‐DAPT scores in CAD patients under a DAPT after a PCI and by HAS‐BLED score developed in AF patients under oral anticoagulation.^21^, ^22^, ^23^ These scores underwent receiver operator curve (ROC)‐analysis for adverse outcomes in COVID‐19 as they include several risk factors, for example, smoking status and obesity, which may be of relevance in the course of COVID‐19.

After addressing the available risk scores our further goal was to generate risk assessment models including cardiovascular comorbidities, cardiac biomarkers, and echocardiographic parameters to predict adverse short‐ and midterm outcomes in patients with SARS‐CoV‐2 infection.

## 2 METHODS

### 2.1 Patient cohort and study inclusion

About 441 consecutive patients, hospitalized for at least 24 h and tested positive for SARS‐CoV‐2 at the University Hospital Tübingen, Germany, from February 2020 until January 2021, were enrolled in this retrospective study. A positive polymerase chain reaction (PCR) test for SARS‐CoV‐2 was the only inclusion criterium, irrespective of the reason for hospital admission. Hospitalized patients which were tested positive for SARS‐CoV‐2 in the course of the hospital stay were also included in the study. Written informed consent was obtained wherever possible. Transthoracic echocardiography (TTE) and electrocardiogram were performed in all patients, if considered clinically indicated. Patients were followed up for 30 days, 6, and 12 months after hospital admission for different study endpoints, irrespective of the duration of the hospital stay. Combined endpoint consisted of first manifestation of either ACM and/or venous/arterial thromboembolism. Venous thromboembolic complications were considered as the occurrence of PE, DVT, venous occlusive disease (VOD), and sinus thrombosis, whereas arterial thrombotic events included AMI, ischemic stroke (IS), and peripheral limb ischemia. Disseminated intravascular coagulation (DIC) was defined as a hypercoagulable based on thromboelastographic findings without a diagnosed thrombosis or bleeding complication and also included in the combined endpoint.^24^ VOD manifests usually after hematopoietic cell transplantation as liver failure due to injury to sinusoidal endothelial cells, which is amplified by a local inflammatory response and activation of coagulation and fibrinolytic pathways, causing liver necrosis in severe disease. However, we observed several cases of VOD in COVID‐19 patients without a history of hematopoietic cell transplantation and therefore considered this hypercoagulability state as a potential complication of SARS‐CoV‐2 infection.^25^ Secondary endpoints comprised ACM, mechanical ventilation, venous/arterial thromboembolism, post‐COVID‐19 syndrome, myocarditis, ACS, heart failure, and rhythm events. We defined post‐COVID‐19 syndrome as a compilation of signs and symptoms that develop during or after an infection consistent with COVID‑19, continue for more than 12 weeks and are not explained by an alternative diagnosis, according to National Institute for Health and Care Excellence.^26^ No patients were lost in the initial 30‐day follow‐up and 48 patients (10.9%) were lost during the later follow‐up up to 12 months.

The study was approved by the institutional ethics committee (238/2018BO2) and complies with the declaration of Helsinki and the good clinical practice guidelines.^27^, ^28^, ^29^

### 2.2 Diagnosis of SARS‐CoV‐2 infection and acute respiratory distress syndrome

SARS‐CoV‐2 virus was detected from nasopharyngeal secretions using a real‐time reverse transcriptase PCR. Acute respiratory distress syndrome (ARDS) was diagnosed based on the Berlin Definition of ARDS.^30^

### 2.3 Imaging and laboratory diagnostics

Chest X‐ray and/or a thoracic computed tomography were performed in symptomatic SARS‐CoV‐2 patients (*n* = 374, 84.8%) and TTE was available in 266 (60.3%) patients in the course of the hospital stay. Peripheral venous blood was drawn for laboratory parameters in the first 24 h after the positive test for SARS‐CoV‐2, if available. Certain laboratory parameters were, however, available only in the further course of disease and the first available values were included in the analysis.

### 2.4 Calculation of selected risk scores

Available risk scores for assessment of ACM and/or myocardial infarction (MI) (DAPT, GRACE 2.0, PARIS‐CTE, PREDICT‐STABLE), ischemic stroke (CHA_2_‐DS_2_‐VASc), and bleeding complications (HAS‐BLED, PARIS‐MB, PRECISE‐DAPT) were calculated in the study cohort.^16^, ^17^, ^18^, ^19^, ^20^, ^21^, ^22^, ^23^

Online calculators for, GRACE 2.0, PRECISE‐DAPT, and DAPT scores are available at https://www.outcomes-umassmed.org/grace/acs_risk2/index.html, http://www.precisedaptscore.com/predapt/webcalculator.html, and http://tools.acc.org/DAPTriskapp/#!/ content/calculator/, respectively. Risk for intrahospital mortality was evaluated by GRACE 2.0 score calculator. CHA_2_‐DS_2_‐VASc, HAS‐BLED, PREDICT‐STABLE, and PARIS scores were calculated manually using corresponding definitions of risk factors.

### 2.5 Statistical analysis

Statistical analysis was performed using IBM SPSS Statistics, version 26.0. Continuous variables are presented as mean values (±standard deviation) and compared using Student's *t*‐test. If necessary, log transformation was applied to achieve normal distribution. Categorical variables were compared using cross‐tabulations and Chi‐square tests. Discriminatory performance of risk scores was evaluated using ROCs and expressed as area under the curve with 95% CI. Depending on the area under the curve (AUC), predictive performance of the risk assessment models was considered very poor (<0.5), poor (0.5.–0.7), good (>0.7), very good (>0.8), or excellent (>0.9). For newly established scores, the leave‐one‐out method (including forward variable selection, inclusion *p* = .05, exclusion *p* = .10) was applied to avoid overoptimism. Established scores were analyzed without further correction. Multiple imputations were applied for missing predictor variables. Thus, within each of the 100 imputations, 411 leave‐one‐out steps were performed using variable selection which resulted in 41 100 different models. Proposed risk assessment scores were obtained from the averaged imputation samples, the AUCs were obtained by averaging results from 100 imputations, standard errors were obtained using Rubin's formula.

## 3 RESULTS

The demographic and clinical characteristics of the patient cohort are listed in Table 1.

**Table 1:** Baseline characteristics of the study cohort

	All (*n* = 441)
Male, *n* (%)	250 (56.7), *n* = 441
Age, years, mean (±SD)	67.0 (±16.4), *n* = 441
BMI, mean (±SD)	28.3 (±5.8), *n* = 299
*Cardiovascular risk factors and comorbidities*	
Arterial hypertension	277/441 (62.8)
Diabetes mellitus, *n* (%)	100/441 (22.7)
Current smoking, *n* (%)	21/441 (4.8)
Hyperlipidemia, *n* (%)	101/441 (22.9)
Positive family history, *n* (%)	11/441 (2.5)
Coronary artery disease, *n* (%)	81/441 (18.4)
Prior myocardial infarction, *n* (%)	45/437 (10.3)
Atrial fibrillation, *n* (%)	108/441 (24.5)
Prior ischemic stroke, *n* (%)	50/441 (11.3)
Chronic kidney disease, *n* (%)	60/441 (13.6)
Chronic obstructive pulmonary disease, *n* (%)	22/441 (5.0)
Obesity, *n* (%)	105/439 (23.9)
*Echocardiographic parameters*	
LVEF, %, mean (±SD)	56.1 (±8.3), *n* = 266
TAPSE, mm, mean (±SD)	22.5 (±6.0), *n* = 187
Impaired right ventricular function, *n* (%)	28 (11.2), *n* = 249
Tricuspid valve insufficiency >1, *n* (%)	30 (12.7), *n* = 237
sPAP, mmHg + CVP mean (±SD)	27.3 (±11.1), *n* = 165
*Radiologic parameters*	
Bilateral/focal infiltrates, *n* (%)	253 (57.4), *n* = 374
*Laboratory parameters*	
Hemoglobin, g/dl, median (25th−75th percentile)	12.7 (10.8‐13.8), *n* = 438
White blood cells, 1/µl, median (25th−75th percentile)	6835.0 (4877.0‐9637.0), *n* = 438
Lymphocytes, 10^3^/µl, median (25th−75th percentile)	0.8 (0.6‐1.2), *n* = 396
Platelets, 10^3^/µl, median (25th−75th percentile)	193 (147‐259), *n* = 438
Creatinine, mg/dl, median (25th−75th percentile)	0.9 (0.7‐1.3), *n* = 430
GFR, ml/m^2^, median (25th−75th percentile)	75.4 (47.8‐99.6), *n* = 428
CRP, mg/dl, median (25th−75th percentile)	6.7 (1.9‐14.6), *n* = 435
Procalcitonin, ng/ml, median (25th−75th percentile)	0.2 (0.1‐0.6), *n* = 357
IL‐6, ng/l, median (25th−75th percentile)	31.4 (14.3‐109.9), *n* = 184
d‐dimer, µg/ml, median (25th−75th percentile)	1.3 (0.8‐3.4), *n* = 344
Troponin‐I, ng/l, median (25th−75th percentile)	16.0 (6.0‐49.0), *n* = 303
NT‐pro‐BNP, ng/l, median (25th−75th percentile)	616.0 (164.0‐3067.0), *n* = 267
Creatine kinase, U/l, median (25th−75th percentile)	113.5 (62.0‐258.3), *n* = 402
Lactate, mmol/l, median (25th−75th percentile)	1.2 (0.9‐1.8), *n* = 323
*Medication at admission*	
ACE inhibitors, *n* (%)	116/431 (26.9)
ARB, *n* (%)	114/431 (26.5)
Aldosterone antagonists, *n* (%)	39/431 (9.0)
Diuretics, *n* (%)	169/430 (39.3)
Calcium channel blockers, *n* (%)	97/431 (22.5)
Beta blockers, *n* (%)	174/431 (40.4)
Oral anticoagulants, *n* (%)	79/431 (18.3)
ASA, *n* (%)	93/431 (21.6)
P2Y12 receptor inhibitors, *n* (%)	16/431 (3.7)
*Medication at discharge*	
ACE inhibitors, *n* (%)	89/350 (25.4)
ARB, *n* (%)	103/350 (29.4)
Aldosterone antagonists, *n* (%)	42/349 (12.0)
Diuretics, *n* (%)	137/350 (39.1)
Calcium channel blockers, *n* (%)	89/350 (25.4)
Beta blockers, *n* (%)	160/349 (45.8)
Oral anticoagulants, *n* (%)	105/350 (30.0)
ASA, *n* (%)	75/348 (21.6)
P2Y12 receptor inhibitors, *n* (%)	16/347 (4.6)

Abbreviations: ACE, angiotensin‐converting factor; ARB, angiotensin receptor blocker; ASA, acetylsalicylic acid; BMI, body mass index; CRP, C‐reactive protein; CVP, central venous pressure; GFR, glomerular filtration rate; IL‐6, interleukin‐6; LVEF, left ventricular ejection fraction; NT‐pro‐BNP, N‐terminal prohormone of brain natriuretic peptide; sPAP, systolic pulmonary artery pressure; TAPSE, tricuspid annular plane systolic excursion.

The distribution of the study endpoints at 30 days, 6, and 12 months after hospital admission is shown in Table 2. Thromboembolic complications consisted of PE (4.1%), DVT (2.3%), VOD (5.9%), and other thrombotic events (1.6%): One sinus thrombosis (0.23%), two cases of disseminated intravascular coagulation with hypercoagulable state (0.45%), one AMI (0.23%), two IS (0.45%), and one lower limb ischemia (0.23%).

**Table 2:** Incidence of the combined endpoint and secondary endpoints

Study endpoints	All (*n* = 441)
**Combined endpoint, *n* (%)**	127 (28.8)
**All‐cause mortality, *n* (%)**	94 (21.3)
**Thromboembolism, *n* (%)**	61 (13.8)
PE, *n* (%)	18 (4.1)
DVT, *n* (%)	10 (2.3)
VOD, *n* (%)	26 (5.9)
Other, *n* (%)	7 (1.6)
**Mechanical ventilation, *n* (%)**	120 (27.2)
**Myocarditis, *n* (%)**	2 (0.5)
**Acute coronary syndrome, *n* (%)**	17 (3.9)
**Heart failure, *n* (%)**	11 (2.5)
**Rhythm event, *n* (%)**	34 (7.7)
**Post‐COVID‐19 syndrome, *n* (%)**	85 (19.3)

Abbreviations: DVT, deep vein thrombosis; PE, pulmonary embolism; VOD, venous occlusive disease.

Table 3 summarizes the discriminatory performance (AUC with 95% CI) of selected existing risk assessment models within a 30‐day follow‐up.

**Table 3:** Discrimination performance of selected pre‐existing risk assessment scores

	AUC (95% CI)							
	CHA2DS2‐VASc	DAPT	GRACE 2.0	HAS‐BLED	PARIS‐CTE	PARIS‐MB	PRECISE‐DAPT	PREDICT‐ STABLE
**Combined endpoint**	0.573 (0.512–0.633)	0.494 (0.430–0.557)	**0.713 (0.637–0.790)**	0.594 (0.537–0.651)	0.574 (0.503–0.646)	0.621 (0.551–0.691)	0.671 (0.606–0.736)	0.595 (0.520–0.671)
**ACM**	0.628 (0.564–0.691)	0.459 (0.386–0.532)	**0.751 (0.675–0.828)**	0.630 (0.569–0.691)	0.605 (0.527–0.684)	0.648 (0.570–0.725)	**0.701 (0.629–0.773)**	0.606 (0.527–0.685)
**Thromboembolism**	0.431 (0.353–0.509)	0.576 (0.499–0.653)	0.541 (0.434–0.648)	0.518 (0.442–0.593)	0.503 (0.412–0.594)	0.565 (0.479–0.652)	0.573 (0.486–0.660)	0.538 (0.431–0.644)
**Mechanical ventilation**	0.475 (0.414–0.537)	0.606 (0.545–0.666)	0.573 (0.494–0.652)	0.543 (0.484–0.603)	0.542 (0.472–0.613)	0.512 (0.442–0.583)	0.540 (0.473–0.607)	0.543 (0.468–0.618)

*Note*: Numbers in bold indicate AUC > 0.7.

Abbreviations: ACM, all‐cause mortality; AUC, area under the curve, CI, confidence interval.

Supporting Information: Table S1 represents demographic and clinical parameters and their univariable discriminatory performance for combined and secondary study endpoints at 30 days.

**Figure 1:**
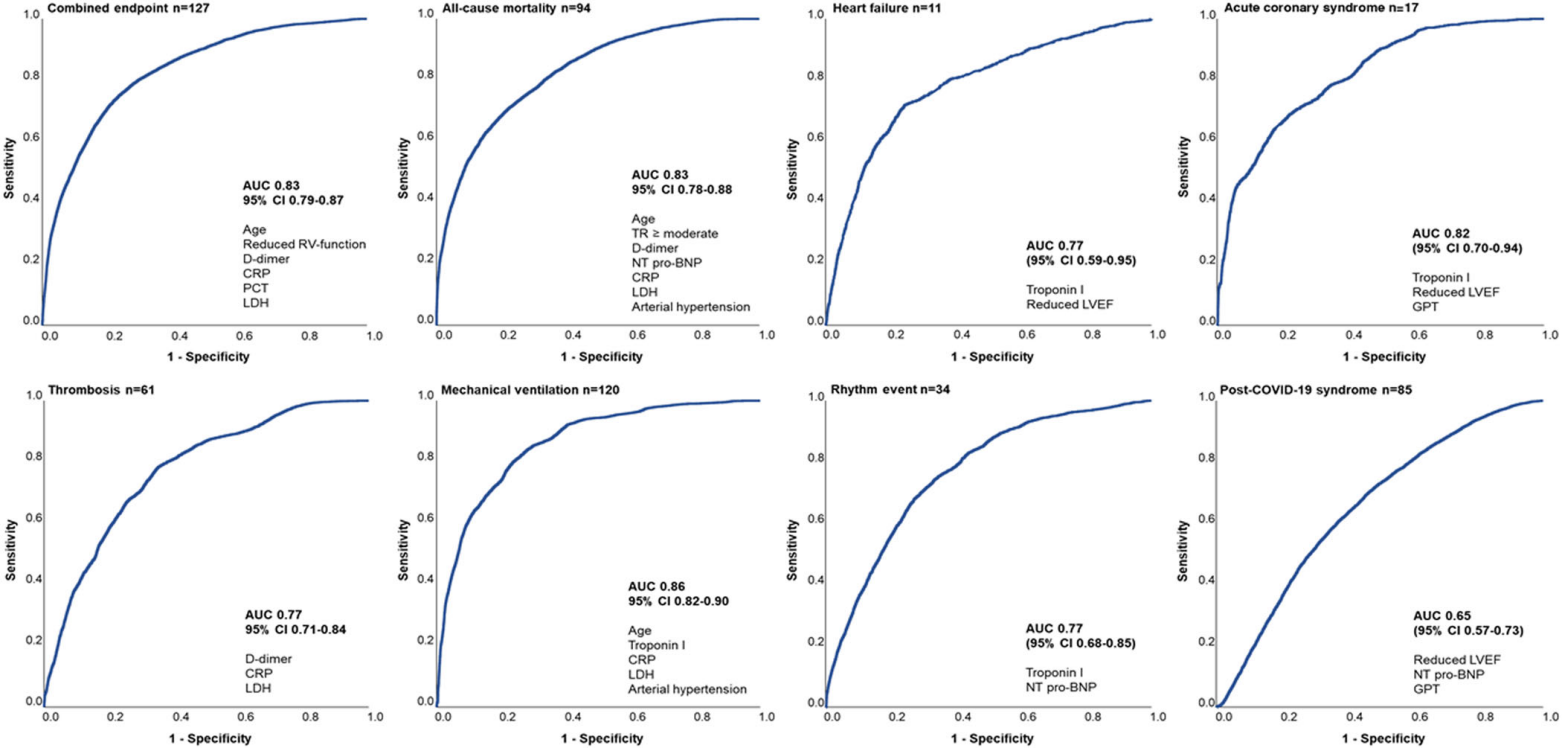
Novel risk assessment models and their discriminatory performance in predicting combined and secondary study endpoints in a short‐ and midterm follow‐up

Figure 1 depicts newly generated risk assessment models which show good (AUC > 0.7) and very good (AUC > 0.8) predictive performance for combined and secondary endpoints within a 30‐day follow‐up. Combined endpoint was best predicted by a model including d‐dimer, CRP, procalcitonin, LDH, age, and reduced RV‐function (AUC = 0.83, 95% CI 0.79–0.87). ACM was best predicted by a combination of d‐dimer, CRP, LDH, NT pro‐BNP, age, moderate/severe TR, and arterial hypertension (AUC = 0.83, 95% CI 0.78–0.88). A combination of three laboratory parameters (d‐dimer, CRP, LDH) best predicted venous/arterial thromboembolism (AUC = 0.77, 95% CI 0.71–0.84). A model including CRP, LDH, troponin I, age, and arterial hypertension reached an AUC = 0.86, 95% CI 0.82–0.90, when predicting mechanical ventilation. Another three risk assessment models could successfully predict myocarditis (AUC of 0.98 [95% CI 0.96 to <1.0]), ACS (AUC 0.82, 95% CI 0.70‐0.94), heart failure (AUC 0.77, 95% CI 0.59–0.95), and rhythm events (AUC 0.77, 95% CI 0.68–0.85). The occurrence of post‐COVID‐19 syndrome could be predicted with an AUC = 0.65, 95% CI 0.57–0.73. The observed sensitivity of selected scores is represented in Supporting Information: Table S2.

Table 4 represents the parameters included in generated risk assessment models and multiplication coefficients needed for risk score calculation.

**Table 4:** Parameters included in generated risk assessment models and their multiplication coefficients

	Combined endpoint	ACM	Venous/arterial thromboembolism	Mechanical ventilation	ACS	Heart failure	Rhythm event	Post‐COVID‐19 syndrome
d‐dimer	1.067	0.647	0.738	–	–	–	–	
CRP	1.033	1.019	1.394	2.478	–	–	–	
Procalcitonin	0.486	–	–	–	–	–	–	
LDH	2.464	2.741	1.648	3.108	–	–	–	
NT pro‐BNP	–	0.611	–	–	–	–	0.806	−0.389
GPT	–	–	–	–	−2.005	–	–	1.004
Troponin I				0.839	1.172	0.996	0.885	–
Age	0.031	0.050	–	−0.030	–	–	–	
Reduced LVEF				–	1.658	2.458	–	−1.181
Reduced RV‐function	1.489	–	–	–	–	–	–	
Moderate/severe TR	–	1.224	–	–	–	–	–	
Arterial hypertension	–	−0.701	–	0.613	–	–	–	

Abbreviations: CRP, C‐reactive protein; GPT, Glutamat‐Pyruvat‐Transaminase; LDH, lactate dehydrogenase; LVEF, left ventricular ejection fraction; NT pro‐BNP, N‐terminal prohormone of brain natriuretic peptide; RV, right ventricular; TR, tricuspid regurgitation.

## 4 DISCUSSION

The main findings of the present study are: (1) risk scores originally established for CAD patients showed better prediction performance for combined endpoint and ACM compared to thromboembolism and mechanical ventilation in the current cohort; (2) GRACE 2.0 score showed best discrimination performance for combined endpoint and ACM when compared to other selected risk assessment models; (3) risk scores including age, reduced left or right ventricular function, moderate/severe tricuspid regurgitation, systolic pulmonary artery pressure, arterial hypertension, d‐dimer, CRP, PCT, TnI, LDH, and NT pro‐BNP, GPT could successfully predict combined endpoint, ACM, venous/arterial thromboembolism and need for mechanical ventilation in a 30‐day follow‐up, whereas myocarditis, ACS, heart failure, rhythm events in a 12‐month follow‐up; (4) prediction of occurrence of the post‐COVID‐19 syndrome in our cohort was poor.

Our study aimed to evaluate the predictive performance of pre‐existing cardiovascular risk assessment models and to generate risk assessment scores, including cardiovascular parameters, to predict ACM, venous/arterial thromboembolism, and/or mechanical ventilation in a short‐term follow‐up of 30 days in a cohort of 441 COVID‐19 patients. We also aimed to generate risk assessment models which could predict the occurrence of post‐COVID‐19 syndrome in a 6‐month follow‐up and cardiovascular complications (myocarditis, ACS, heart failure, rhythm events) in a 12‐month follow‐up.

CVRFs and comorbidities are associated with increased mortality and worse outcomes in COVID‐19.^2^, ^3^ In the current cohort, arterial hypertension, diabetes mellitus type 2, hyperlipidemia and obesity were the most prevalent risk factors/comorbidities. As the link between CVD and COVID‐19 is well‐established, we postulated, that risk assessment scores developed in CAD patients could be of use for risk assessment in individuals suffering from COVID‐19. Therefore, we evaluated the discriminatory performance of selected scores for predicting adverse outcomes. DAPT, GRACE 2.0, PARIS‐CTE, PREDICT‐STABLE, CHA2‐DS2‐VASc, HAS‐BLED, PARIS‐MB, PRECISE‐DAPT scores were originally generated for prediction of ACM, cardiovascular ischemic and bleeding complications and include various cardiovascular risk factors and comorbidities as predictive parameters. In general, all established scores performed better in the prediction of the combined endpoint and ACM compared to thromboembolic complications and need for mechanical ventilation. However, GRACE 2.0 score was the only score to show good (AUC > 0.7) discrimination performance for the combined endpoint and ACM within the 30‐day follow‐up period, whereas thromboembolic complications and mechanical ventilation were predicted poorly. GRACE 2.0 score combines markers of myocardial ischemia (electrocardiographic changes, elevated troponin) and heart failure with universal vital/laboratory parameters, for example, heart rate, systolic blood pressure, and creatinine. Adding the latter might have led to better prediction of mortality compared to scores including mainly CVRFs and/or cardiac comorbidities e.g. DAPT and PARIS‐CTE.

To identify potential parameters which could be included in risk assessment models for COVID‐19, we performed ROC‐analyses with laboratory markers, echocardiographic parameters, and CVRFs in our COVID‐19 cohort. Laboratory parameters (cardiac and inflammatory markers) showed good (AUC > 0.7) predictive performance for combined endpoint, ACM, venous/arterial thromboembolism and mechanical ventilation in a short‐term follow‐up, and cardiovascular complications (myocarditis, ACS, heart failure, and rhythm events) in a 12‐month follow‐up, whereas independent cardiovascular risk factors/comorbidities and echocardiographic parameters failed to discriminate the study endpoints. However, combining laboratory with demographic, echocardiographic parameters, and cardiovascular risk factors/comorbidities revealed several models with very good (AUC > 0.8) discrimination of combined endpoint, ACM, mechanical ventilation, myocarditis, and ACS, and good (AUC > 0.7) predictive performance for venous/arterial thromboembolism, heart failure, and rhythm events. However, the predictive performance of myocarditis should be interpreted with caution due to a very low number (*n* = 2) of events observed in our cohort. The generated risk assessment models included age, reduced left ventricular ejection fraction, reduced right ventricular function, moderate/severe tricuspid regurgitation, systolic pulmonary artery pressure, arterial hypertension, d‐dimer, CRP, PCT, TnI, LDH, GPT, and/or NT pro‐BNP. In the context of other risk assessments scores developed for COVID‐19 patients, our risk models showed similar prediction performance. Compared to the 4C Mortality Score based on a cohort of over 57 000 patients in the United Kingdom and developed for prediction of intra‐hospital mortality (AUC = 0.79) our model predicted ACM at 30 days slightly better (AUC = 0.827).^12^ However, Quick COVID‐19 Severity Index (qCSI), could predict 24‐h ACM and ICU admission slightly better, compared to our model for ACM (AUC= 0.89 vs. AUC = 0.827).^10^ Another score, COVID‐GRAM, which predicts a combined endpoint including admission at ICU, invasive ventilation, and/or death reached an AUC = 0.88, whereas our models predicted both ACM and mechanical ventilation almost as well (AUC = 0.827 and 0.862, respectively).^11^

As associations between cardiac comorbidities and poor COVID‐19 prognosis are evident, we expected CVRFs to have significant influence on outcomes. In our cohort, however, only arterial hypertension was identified as a significant predictor of ACM and mechanical ventilation. A score by Galloway et al. and VACO‐index also identified diabetes mellitus as a good discriminator for prediction of ICU admission and ACM.^13^, ^14^ To the best of our knowledge, none of the risk assessment models developed for prediction of adverse outcomes in COVID‐19 include specific myocardial biomarkers, for example, troponin I, NT pro‐BNP, and echocardiographic parameters.

The finding, that beyond inflammatory parameters also cardiac biomarkers and echocardiographic parameters may serve as significant predictors of poor outcomes corresponds to current evidence suggesting that right ventricular dysfunction is associated with worse prognosis in COVID‐19.^15^ Left ventricular ejection fraction or heart failure with reduced ejection fraction, however, showed poor performance as individual discriminators in predicting COVID‐19 outcomes in our cohort. This supports the fact that right heart failure caused by ARDS and/or PE is a driving force leading to worse outcomes in COVID‐19.

## 5 CONCLUSION

As cardiovascular risk factors and comorbidities are highly prevalent in COVID‐19 and associated with poor prognosis, we aimed to establish risk assessment models including cardiovascular biomarkers, risk factors, and echocardiographic parameters to predict adverse short‐ and midterm outcomes in COVID‐19. Risk scores including laboratory, demographic, as well as echocardiographic parameters and cardiovascular risk factors showed markedly better performance in prediction of ACM, venous/arterial thromboembolism and need for mechanical ventilation in COVID‐19 compared to established cardiovascular risk assessment models.

## 6 LIMITATIONS

Limitations of this study include the moderately sized patient cohort used for generation of the risk assessment models. Patients included in the study cohort were potentially different in terms of co‐morbidities before SARS‐CoV‐2 infection which could have had a severe impact on their prognosis. A limitation of our study is that we cannot discriminate between pre‐existing or SARS‐CoV‐2‐induced elevated pulmonary artery pressure, heart failure, elevated NT pro‐BNP, and so on. However, even if not directly caused by COVID‐19, parameters included in our models show good discriminatory performance for the pre‐defined endpoints and thus remain, in our opinion, important factors for prognosis in COVID‐19 patients. Furthermore, this is a retrospective single‐center study that lacks an external validation cohort. As the vaccination against COVID‐19 started in January 2021 and we recruited patients for the current study in the period from February 2020 until January 2021, we cannot deliver information considering the vaccination status of the patients included in the study. Finally, not all of the parameters included in the study were available for all patients.

## AUTHOR CONTRIBUTIONS

Monika Zdanyte: Data collection, statistical analysis, drafting of the manuscript. Peter Martus: Expert statistical analysis, critical revision of the manuscript. Jeremy Nestele, Alexander Bild, Lars Mizera, Andreas Glatthaar, Álvaro Petersen Uribe, Frederic Emschermann, and Jessica‐Kristin Henes: Data collection, critical revision of the manuscript. Tobias Geisler, Karin Müller, and Meinrad Gawaz: Critical revision of the manuscript. Dominik Rath: Study concept, data collection, critical revision of the manuscript.

## CONFLICT OF INTEREST

The authors declare no conflicts of interest.

## Supporting information

Supporting information.

## Data Availability

The data that support the findings of this study are openly available under request.
