# Supplementary material for: Risk assessment in COVID‐19: Prognostic importance of cardiovascular parameters
Source: Clin Cardiol. 2022 Jul 5;45(9):943–51. doi: 10.1002/clc.23883 (PMC9350294; doi:10.1002/clc.23883)
Supplement: Supplementary file 1 — Supporting information. [file CLC-45-943-s001.docx]

**Supplementary Table 1: Discrimination performance of demographic and clinical parameters**

|  | **Combined endpoint** | **ACM** | **Venous/arterial thromboembolism** | **Mechanical ventilation** |
| --- | --- | --- | --- | --- |
| **AUC (95% CI)** | | | | |
| **Age** | 0.616  (0.557-0.674) | 0.688  (0.627-0.749) | 0.455  (0.384-0.525) | 0.473  (0.415-0.532) |
| **Male gender** | 0.561  (0.502-0.619) | 0.561  (0.498-0.624) | 0.613  (0.541-0.684) | 0.611  (0.556-0.666) |
| **Current smoking** | 0.494 (0.435-0.554) | 0.483 (0.419-0.548) | 0.501 (0.423-0.579) | 0.479 (0.419-0.538) |
| **Arterial hypertension** | 0.523  (0.464-0.583) | 0.527  (0.462-0.592) | 0.507  (0.429-0.584) | 0.567  (0.508-0.625) |
| **Diabetes mellitus** | 0.523  (0.463-0.583) | 0.532  (0.465-0.599) | 0.502  (0.423-0.580) | 0.550  (0.489-0.612) |
| **Hyperlipidemia** | 0.488  (0.429-0.548) | 0.496  (0.431-0.562) | 0.500  (0.422-0.578) | 0.480  (0.420-0.540) |
| **BMI** | 0.541  (0.472-0.611) | 0.508  (0.432-0.584) | 0.532  (0.443-0.621) | 0.613  (0.549-0.676) |
| **CCS** | 0.535  (0.475-0.596) | 0.554  (0.486-0.622) | 0.506  (0.427-0.585) | 0.526  (0.465-0.588) |
| **CAD** | 0.526  (0.466-0.586) | 0.546  (0.478-0.613) | 0.498  (0.420-0.576) | 0.517  (0.456-0.578) |
| **CAD severity** | 0.516  (0.455-0.578) | 0.534  (0.465-0.604) | 0.488  (0.408-0.567) | 0.520  (0.457-0.582) |
| **Heart failure** | 0.550  (0.487-0.612) | 0.566  (0.496-0.636) | 0.531  (0.449-0.612) | 0.537  (0.474-0.599) |
| **CKD** | 0.537  (0.476-0.598) | 0.556  (0.487-0.624) | 0.516  (0.437-0.596) | 0.515  (0.454-0.576) |
| **COPD** | 0.493  (0.433-0.552) | 0.495  (0.430-0.561) | 0.481  (0.405-0.557) | 0.494  (0.434-0.555) |
| **Malignancy** | 0.550  (0.489-0.611) | 0.544  (0.476-0.612) | 0.543  (0.461-0.624) | 0.506  (0.445-0.567) |
| **Asthma** | 0.492  (0.433-0.551) | 0.479  (0.415-0.544) | 0.513  (0.434-0.592) | 0.493  (0.433-0.554) |
| **Prior immunosuppression** | 0.522  (0.462-0.582) | 0.508  (0.442-0.575) | 0.551  (0.470-0.632) | 0.484  (0.423-0.544) |
| **Creatinine** | 0.617  (0.556-0.678) | 0.635  (0.569-0.702) | 0.571  (0.485-0.656) | 0.581  (0.520-0.641) |
| **Hemoglobin** | 0.353  (0.296-0.411) | 0.382  (0.317-0.447) | 0.390  (0.315-0.465) | 0.418  (0.358-0.477) |
| **Leukocytes** | 0.633  (0.575-0.692) | 0.613  (0.547-0.679) | 0.638  (0.557-0.718) | 0.638  (0.578-0.698) |
| **Lymphocytes** | 0.359  (0.298-0.420) | 0.345  (0.279-0.411) | 0.379  (0.297-0.462) | 0.375  (0.314-0.435) |
| **Platelets** | 0.454  (0.395-0.512) | 0.447  (0.384-0.511) | 0.463  (0.384-0.542) | 0.525  (0.462-0.588) |
| **D-dimer** | **0.730**  **(0.674-0.786)** | 0.681  (0.615-0.747) | 0.697  (0.628-0.767) | 0.667  (0.608-0.726) |
| **GFR** | 0.386  (0.323-0.449) | 0.367  (0.299-0.435) | 0.450  (0.361-0.539) | 0.440  (0.378-0.502) |
| **CRP** | **0.763**  **(0.713-0.812)** | **0.723**  **(0.667-0.780)** | **0.748**  **(0.681-0.816)** | **0.820**  **(0.777-0.862)** |
| **Procalcitonin** | **0.735**  **(0.680-0.790)** | **0.724**  **(0.663-0.784)** | 0.684  (0.609-0.759) | **0.748**  **(0.695-0.800)** |
| **IL-6** | **0.764**  **(0.691-0.837)** | **0.719**  **(0.638-0.800)** | **0.702**  **(0.606-0.798)** | **0.737**  **(0.663-0.810)** |
| **Troponin I** | **0.711**  **(0.649-0.772)** | **0.725**  **(0.662-0.787)** | 0.626  (0.541-0.710) | 0.695  (0.635-0.755) |
| **NT pro-BNP** | **0.740**  **(0.676-0.803)** | **0.786**  **(0.723-0.849)** | 0.600  (0.507-0.693) | 0.643  (0.569-0.717) |
| **LDH** | **0.714**  **(0.656-0.771)** | 0.696  (0.632-0.760) | 0.687  (0.610-0.764) | **0.783**  **(0.733-0.833)** |
| **Lactate** | 0.598  (0.532-0.663) | 0.597  (0.524-0.670) | 0.533  (0.453-0.614) | 0.561  (0.495-0.627) |
| **pH** | 0.406  (0.337-0.474) | 0.425  (0.346-0.504) | 0.443  (0.356-0.530) | 0.416  (0.348-0.485) |
| **Systolic LVEF** | 0.390  (0.312-0.468) | 0.353 (0.264-0.441) | 0.460  (0.361-0.559) | 0.424  (0.346-0.502) |
| **TR > mild** | 0.589  (0.506-0.671) | 0.607  (0.512-0.701) | 0.526  (0.420-0.633) | 0.524  (0.438-0.609) |
| **sPAP** | 0.601  (0.508-0.694) | 0.590  (0.486-0.694) | 0.598  (0.476-0.720) | 0.533  (0.428-0.637) |
| **Reduced RV-function** | 0.597  (0.517-0.677) | 0.600  (0.510-0.691) | 0.529  (0.425-0.633) | 0.534  (0.452-0.616) |
| **RV dilatation** | 0.504  (0.426-0.583) | 0.517  (0.429-0.604) | 0.476  (0.377-0.576) | 0.568  (0.486-0.650) |
| **TAPSE** | 0.426  (0.336-0.517) | 0.437  (0.335-0.540) | 0.508  (0.395-0.621) | 0.471  (0.382-0.560) |

*Abbreviations: BMI (body mass index), CCS (chronic coronary syndrome), CAD (coronary artery disease), CKD (chronic kidney disease), COPD (chronic obstructive pulmonary disease), GFR (glomerular filtration rate), CRP (C-reactive protein), IL-6 (interleukin-6), NT-pro-BNP (N-terminal prohormone of brain natriuretic peptide), LDH (lactate dehydrogenase), LVEF (left ventricular ejection fraction), TR (tricuspid regurgitation), sPAP (systolic pulmonary artery pressure), RV (right-ventricular), TAPSE (tricuspid annular plane systolic excursion). (Numbers in bold indicate AUC>0.7)*

**Supplementary Table 2: Observed sensitivity and specificity of generated risk assessment models**

| **Sensitivity** | **Combined endpoint** | **ACD** | **Venous/arterial thromboembolism** | **Mechanical ventilation** | **ACS** | **Heart failure** | **Rhythm event** | **Post-COVID-19 syndrome** |
| --- | --- | --- | --- | --- | --- | --- | --- | --- |
| 80% | 70% | 67% | 62% | 76% | 78% | 90% | 62% | 55% |
| 90% | 51% | 51% | 38% | 62% | 58% | 58% | 49% | 42% |
| 95% | 38% | 38% | 29% | 46% | 26% | 14% | 27% | 28% |
